# Modeling method for refraction error compensation in ground object detection

**DOI:** 10.1371/journal.pone.0339265

**Published:** 2026-01-27

**Authors:** Daochun Yu, Zhen Chen

**Affiliations:** 1 School of Physics and Electronic Engineering, Jining University, Qufu, China; 2 Department of Clinical Medical Engineering, The First Affiliated Hospital with Nanjing Medical University/Jiangsu Province Hospital, Nanjing, Jiangsu, China; King Mongkut’s University of Technology North Bangkok, THAILAND

## Abstract

The refraction error compensation model for ground object detection is established. Firstly, based on a 100-meter interval, the area between the aircraft and the ground objects is stratified, and the Hopfield model of the atmospheric refractive index is combined with the e-index model to achieve an accurate simulation of the atmospheric refractive index profile. Then, based on the information such as the line-of-sight direction, flight altitude, and observation wavelength of the ground object observation target by the aircraft, combined with Snell’s law, the distance from the projection of the aircraft on the Earth’s surface to the true position of the ground object is given, and the true elevation angle is derived. Finally, by calculating the difference between the apparent elevation angle and the true elevation angle of the aircraft, the elevation angle error model of the aircraft is established to correct the elevation angle error of the aircraft caused by atmospheric refraction and is applied to the precise identification and positioning of the observed target.

## 1 Introduction

The density of the Earth’s atmosphere is a continuous curve that varies with altitude. When observing a feature target from an aircraft, because the light has to traverse the atmosphere with different concentrations, the light is bent by refraction, which ultimately leads to a difference between the observed feature position and the actual situation, thus affecting the detection and localization accuracy of the surface target [1,2]. The elevation angle error of atmospheric refraction eventually leads to the distance error between the observed and actual positions of the feature target, and it has been shown that when the observed zenith angle is $30^{\circ }$ and $60^{\circ }$, the distance error between the true position and the apparent position of the feature due to atmospheric refraction reaches 2.22 m and 17.85 m, respectively [3]. Therefore, reducing the elevation angle error is of great value to improve the positioning accuracy of the detected target.

Astronomical atmospheric refraction has been widely studied in the last 20 years, such as the widely used Pulkovo atmospheric refraction table, which corrects for bending angle errors in astronomical observations, thus improving the accuracy of astronomical observations [4,5]. Starlight refraction navigation, as a spacecraft autonomous navigation method, the high-precision modeling of the starlight refraction angle when the apparent zenith distance is $90^{\circ }$ is an important factor affecting the navigation accuracy, which is also an application of the theory of astronomical atmospheric refraction [6]. In the field of Earth observation and remote sensing detection, the elevation angle error compensation technique is less studied, and the technique is less mature compared to the astronomical atmospheric refraction correction technique [7]. Synthetic aperture radar (SAR) has important applications in natural disaster monitoring, environmental quality monitoring, ocean monitoring, resource exploration, crop yield estimation, and surveying and mapping, and in order to improve the accuracy of the earth observation, the elevation error correction is particularly important [8–10]. In most of the current researches to improve the SAR positioning accuracy, it is mainly realized by designing the corresponding mechanical structure rather than compensating the elevation error [11]. For the problem of SAR remote sensing image distortion, most of the researches are mainly realized by developing corresponding image processing algorithms instead of compensating the elevation error to correct the distorted image [12]. Based on the current research status, a refractive error compensation modeling method is proposed for Earth surface detection targets. Firstly, the atmosphere is precisely stratified, and the refraction modeling is completed by combining the atmospheric temperature, pressure and other parameters, then the Hopfield model of atmospheric refractive index and the e-index model are combined to realize the simulation of the atmospheric refractive index contour in the process of construction, and finally the real elevation angle is calculated to get the elevation angle error.

The structure of this article is as follows. The geometric description and modeling process of elevation angle error are described in Sect 2. Sect 3 presents the calculation results of the elevation angle error and the influence of atmospheric parameters on the elevation angle error. Sect 4 discusses the model’s limitations, potential applications, and future improvements. Sect 5 presents the conclusion.

## 2 Modeling of elevation angle error

**Fig 1:**
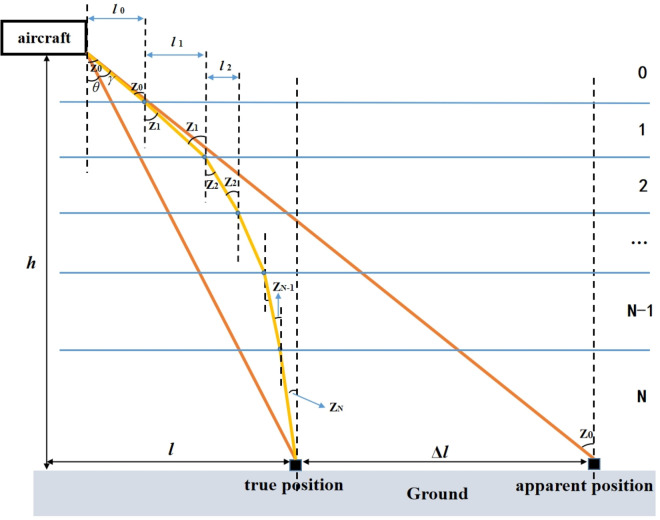
Observational geometry of aircraft’s observation of the Earth.

Light is refracted as it travels through the atmosphere, resulting in a positional deviation between the real position of the feature and the apparent position of the feature, and the angle between the line connecting the aircraft to the real position of the feature target and the line connecting the aircraft to the apparent position of the feature target is defined as the elevation angle error, as shown by *γ* in Fig 1. The apparent and real positions of the observed objects are marked in the figure. Elevation angle error *γ*, real elevation angle *Z*_0_, and apparent elevation angle *Z*_0_ at the observation position are also shown in Fig 1. The apparent and real positions of the observed objects are marked in the figure. The elevation angle error *γ*, as the value to be solved, can be expressed by the following equation,

Fig 1. Observational geometry of aircraft’s observation of the Earth.

$$\gamma =Z_{0}-\theta$$ (1)

where the apparent elevation angle *Z*_0_ (a known quantity) is the angle between the aircraft-apparent object line and the aircraft-Earth center line, and the true elevation angle *θ* (an unknown quantity) is the corresponding angle to the true ground object position. The unknown angle *θ* can be determined by solving Eq 2,

$$\theta =\text{arctan}\frac{l}{h}$$ (2)

where *h* is the distance from the aircraft to the Earth’s surface, which is a known quantity. *l* is the distance from the projection of the aircraft on the Earth’s surface to the true position of the ground object, which is an unknown quantity and needs to be solved to obtain it. The specific solution process is as follows.

Fig 1. Observational geometry of aircraft’s observation of the Earth. The apparent and real positions of the observed objects are marked in the figure. Elevation angle error *γ*, real elevation angle *Z*_0_, and apparent elevation angle *Z*_0_ at the observation position are also shown in the figure.

Firstly, the Earth’s atmosphere is precisely stratified. To ensure the accuracy of the calculation results and the computing speed of the program, the distance between two adjacent atmospheric layers is generally fixed at 100 meters. After verification, when the height resolution is 100 meters, the fluctuation of elevation angle error is relatively small [7]. Suppose the atmospheric density is the same within the same layer, but different between different layers. Then, the Snell’s law is satisfied between adjacent layers. The specific form of Snell’s law is as follows,

$$\begin{array}{c} n_{0}\text{sin}Z_{0}=n_{1}\text{sin}Z_{1}\\ n_{1}\text{sin}Z_{1}=n_{2}\text{sin}Z_{2}\\ n_{2}\text{sin}Z_{2}=n_{3}\text{sin}Z_{3}\\ ......\\ n_{i-1}\text{sin}Z_{i-1}=n_{i}\text{sin}Z_{i}=\text{constant} \end{array}$$ (3)

where *n*_0_ is the refractive index at the observed position, *Z*_0_ is the apparent elevation angle from the observed position, *n*_*i*_ is the refractive index at the *i*th layer, *Z*_*i*_ is the elevation angle of the light at the *i*th atmospheric layer. Suppose the Earth’s atmosphere are divided into *N* layers, then *i*=1,2,3⋯N. Thus, the refraction angle of each layer can be calculated. The specific equation is as follows,

$$\begin{array}{c} Z_{1}=\text{arcsin}\frac{n_{0}\text{sin}Z_{0}}{n_{1}}\\ Z_{2}=\text{arcsin}\frac{n_{1}\text{sin}Z_{1}}{n_{2}}\\ Z_{3}=\text{arcsin}\frac{n_{2}\text{sin}Z_{2}}{n_{3}}\\ ......\\ Z_{i}=\text{arcsin}\frac{n_{i-1}\text{sin}Z_{i-1}}{n_{i}} \end{array}$$ (4)

The horizontal distance between two adjacent atmospheres can be expressed as the following relationship,

$$l_{i}=step*\text{tan}Z_{i}$$ (5)

where *step* represents the vertical distance between two adjacent atmospheres, which is 100 meters. Based on the horizontal distance between every two adjacent layers, the distance *l* between the projection of the aircraft on the ground surface and the true position of the ground object can be obtained,

$$l=\sum_{i=0}^{N}l_{i}$$ (6)

The true elevation angle of the aircraft can be obtained through Eq (2), and then the elevation angle error *γ* caused by atmospheric refraction can be obtained through Eq (1).

The solution of the atmospheric refractive index in Eq (4) is a key point, which is related to the calculation of the refractive angle of each layer of the atmosphere. *n*_0_ represents the atmospheric refractive index of the apparent position of the ground object and can be expressed by the following equation,

$$\left\{ \begin{array}{cc} n_{g}=1+(2876.04+\frac{3\times 16.288}{\lambda^{2}}+\frac{5\times 0.136}{\lambda^{4}})\times 10^{-7} & \\ n=1+\frac{(n_{g}-1)\times P}{101325(1+\alpha t)}-\frac{5.5\times 10^{-8}e}{133.3224(1+\alpha t)} & \end{array}\right.$$ (7)

where *α*=1/273.16, *t*, *P*, *e*, and *λ* respectively refer to the atmospheric temperature, pressure, vapor pressure, and wavelength at the location of the ground object, with units of ^∘^*C*, Pa, Pa, and *μ*m respectively. The refractive index of the atmosphere along the path of light propagation can be expressed by the following equation,

$$n=\left\{ \begin{array}{cc} 1+(n_{0}-1)\cdot 10^{6}\cdot (\frac{h_{d}-h_{i}}{h_{d}-h_{0}}{)}^{4},\;\;\;\;h_{i}\leq h_{d} & \\ 1+(n_{H}-1)\cdot 10^{6}\cdot e^{\beta (h_{i}-h_{d})10^{-4}},\;\;\;\;h_{i}>h_{d} & \end{array}\right.$$ (8)

where *h*_*d*_ represents the elevation, which can be calculated by 40136+148.72×t, with the unit being m. Below the elevation, the refractive index of the atmosphere at each altitude position is given by the Hopfield model, and above the elevation, the refractive index of the atmosphere at each altitude position is given by the e-index model, where *n*_*H*_ represents the refractive index of the atmosphere at the elevation. *β* is the piecewise fitting index. The Hopfield model is employed for altitudes below the elevation (*h*_*d*_) due to its accuracy in modeling the troposphere and stratosphere, where temperature and pressure gradients are significant. The e-index model is used above *h*_*d*_ to account for the exponential decay of atmospheric density in the upper layers [13]. This hybrid approach ensures accuracy across all atmospheric regimes.

In summary, the modeling process detailed in this section effectively constructs the specific form of the unified master equation,

γ=f(t,P,e,λ) (9)

The atmospheric stratification and the application of Snell’s law translate the inputs—*t*, *P*, *e*, and *λ* —into a precise refractive index profile, which is then used to compute the final elevation angle error *γ*. This framework ensures that all critical factors are integrated into a comprehensive error compensation model.

## 3 Analysis of influencing factors of elevation angle error and calculation results

### The influence of vapor pressure on elevation angle error

Although the water vapor content in the atmosphere is not high, it still has an impact on the refractive index, thereby affecting the elevation error. The water vapor pressure has obvious latitudinal distribution characteristics, with a maximum of approximately 30 hPa at the equator and gradually decreasing towards the poles. The variation range of water vapor pressure on the land surface is between 0 and 30 hPa, and it is relatively large near the ground. Then it decreases rapidly with the increase in height. Assuming that the vertical distribution of water vapor pressure is as shown in the following equation,

$$e=e_{0}\cdot e^{-h^{{\;}^{\prime }}}$$ (10)

where *e*_0_ represents the water vapor pressure at the Earth’s surface, and $h^{{\;}^{\prime }}$ indicates the distance above the Earth’s surface, with the unit of km.

In order to analyze the influence of vapor pressure on the elevation angle error, the elevation angle error was calculated respectively under the conditions of considering vapor pressure and not considering vapor pressure, and finally the gap between the two was calculated. The environmental parameters are: the longitude and latitude of Beijing area ($39.56^{\circ }$,116.$20^{\circ }$), time 12:00:00 on September 1, 2022 (UTC), and wavelength 0.58 *μ*m. Calculate the elevation angle errors of the aircraft at flight altitudes of 40 km, 70 km, and 100 km respectively under the conditions of *e*_0_= 30 hPa and *e*_0_= 0 (unit: arcseconds), and calculate the difference ΔE between the two. ΔE is defined as the difference between the elevation angle error under *e*_0_= 0 and the elevation angle error under *e*_0_= 30 hPa. The calculation results are shown in Table 1.

**Table 1:** The elevation Angle errors of aircraft at flight altitudes of 40 kilometers, 70 kilometers, and 100 kilometers under the influence of vapor pressure. (Unit: arcseconds).

Altitude	Elevation angle	$e_{0}=30$ hPa	$e_{0}=0$ hPa	ΔE
40 km	$5^{\circ }$	1.0574	1.0583	5$\times 10^{-4}$
	$10^{\circ }$	2.1319	2.1329	1$\times 10^{-3}$
	$15^{\circ }$	3.2396	3.2412	1.6$\times 10^{-3}$
	$20^{\circ }$	4.4005	4.4026	2.1$\times 10^{-3}$
	$25^{\circ }$	5.6377	5.6404	2.7$\times 10^{-3}$
	$30^{\circ }$	6.9801	6.9834	3.3$\times 10^{-3}$
70 km	$5^{\circ }$	0.6154	0.6157	3$\times 10^{-4}$
	$10^{\circ }$	1.2402	1.2408	6$\times 10^{-4}$
	$15^{\circ }$	1.8847	1.8855	8$\times 10^{-4}$
	$20^{\circ }$	2.5600	2.5612	1.2$\times 10^{-3}$
	$25^{\circ }$	3.2798	3.2813	1.5$\times 10^{-3}$
	$30^{\circ }$	6.0607	4.0626	1.9$\times 10^{-3}$
100 km	$5^{\circ }$	0.4303	0.4305	2$\times 10^{-4}$
	$10^{\circ }$	0.8672	0.8676	4$\times 10^{-4}$
	$15^{\circ }$	1.3178	1.3185	7$\times 10^{-4}$
	$20^{\circ }$	1.7901	1.7909	8$\times 10^{-3}$
	$25^{\circ }$	2.2934	2.2944	1$\times 10^{-3}$
	$30^{\circ }$	2.8394	2.8407	1.3$\times 10^{-3}$

Based on the above calculation results, it is found that the influence of water vapor pressure on the elevation angle error is within 10^−4^ to 10^−3^ arcseconds, and its impact on the elevation angle error accuracy is approximately 0.005%. Therefore, under normal weather conditions, the influence of water vapor pressure on the refraction elevation angle error of ground objects can be ignored.

### The influence of temperature on elevation angle error

The temperature of the Earth’s atmosphere does not change uniformly. In the troposphere and mesosphere, the atmospheric temperature decreases with the increase of altitude. Specifically, in the troposphere, for every 1 km increase in altitude, the temperature drops by approximately 6 ^∘^*C*. In the stratosphere, an inversion layer occurs, where the temperature rises with the increase of altitude. In the thermosphere, the temperature also increases with the increase of altitude. In order to analyze the influence of temperature on the elevation angle error, in practical operation, assuming the pressure remains constant and the vapor pressure e=0, the variation of the elevation angle error under the condition of temperature change ΔT is calculated respectively.

The environmental parameters are as follows: the longitude and latitude of Beijing area ($39.56^{\circ }$,116.$20^{\circ }$), the time is 12:00 UTC on September 1, 2022, the solar activity index F107 is 60 sfu, the geomagnetic activity index Ap is 10 nT, and the wavelength is 0.58 *μ*m. Based on this environmental parameter, the initial values of temperature and pressure at the location of the ground object were calculated using the NRLMSIS 2.0 model. These values can also be given through other models or measured data.

The elevation angle errors (in arcseconds) of the aircraft at flight altitudes of 40 km, 70 km, and 100 km under the conditions of temperature change ΔT (±1 K, ±5 K, ±10 K) were calculated respectively, as shown in Table 2.

**Table 2:** The elevation angle errors of aircraft at flight altitudes of 40 kilometers, 70 kilometers, and 100 kilometers under the influence of temperature. (Unit: arcseconds)

Altitude	Angle	ΔT=−10 K	ΔT=−5 K	ΔT=−1 K	ΔT=0 K	ΔT=1 K	ΔT=5 K	ΔT=10 K
40 km	$5^{\circ }$	1.1012	1.0793	1.0624	1.0583	1.0542	1.0381	1.0186
	$10^{\circ }$	2.2195	2.1753	2.1412	2.1329	2.1246	2.0921	2.0529
	$15^{\circ }$	3.3727	3.3056	3.2538	3.2412	3.2286	3.1792	3.1196
	$20^{\circ }$	4.5813	4.4902	4.4198	4.4026	4.3855	4.3184	4.2375
	$25^{\circ }$	5.8694	5.7526	5.6625	5.6404	5.6185	5.5326	5.4289
	$30^{\circ }$	7.2669	7.1223	7.0108	6.9834	6.9563	6.8499	6.7215
70 km	$5^{\circ }$	0.6407	0.6279	0.6181	0.6157	0.6133	0.6039	0.5926
	$10^{\circ }$	1.2912	1.2655	1.2457	1.2408	1.2360	1.2171	1.1943
	$15^{\circ }$	1.9621	1.9231	1.8929	1.8855	1.8782	1.8495	1.8148
	$20^{\circ }$	2.6652	2.6122	2.5712	2.5612	2.5513	2.5122	2.4651
	$25^{\circ }$	3.4146	3.3466	3.2942	3.2813	3.2686	3.2185	3.1582
	$30^{\circ }$	4.2276	4.1434	4.0785	4.0626	4.0468	3.9849	3.9102
100 km	$5^{\circ }$	0.4480	0.4391	0.4322	0.4305	0.4288	0.4223	0.4143
	$10^{\circ }$	0.9029	0.8849	0.8710	0.8676	0.8643	0.8510	0.8351
	$15^{\circ }$	1.3720	1.3447	1.3236	1.3185	1.3133	1.2932	1.2690
	$20^{\circ }$	1.8637	1.8266	1.7979	1.7909	1.7839	1.7566	1.7237
	$25^{\circ }$	2.3876	2.3401	2.3034	2.2944	2.2855	2.2505	2.2083
	$30^{\circ }$	2.9561	2.8973	2.8518	2.8407	2.8297	2.7864	2.7341

Based on the ground object refraction elevation angle error values in Table 2, calculate the difference error between the elevation angle error under the conditions of ΔT change ±1 K, ±5 K and ±10 K and the elevation angle error under the condition of ΔT being 0 respectively. Here, it is defined as: The difference between the elevation angle error with a ΔT change of ±1 K and the elevation angle error under the condition of ΔT being 0 is *error*1; the difference between the elevation angle error with a ΔT change of ±5 K and the elevation angle error under the condition of ΔT being 0 is *error*5; the difference between the elevation angle error with a ΔT change of ±10 K and the elevation angle error under the condition of ΔT being 0 is *error*10. The absolute values of the *error*1, *error*5, and *error*10 ranges within the 5-$30^{\circ }$ elevation angle range of the aircraft at flight altitudes of 40 km, 70 km, and 100 km respectively are shown in Table 3.

**Table 3:** The absolute values of the ranges of *error*1, *error*5, and *error*10.

Altitude	*|error1|*	*|error5|*	*|error10|*
40 km	0.0041∼0.0274	0.0202∼0.1389	0.0397∼0.2835
70 km	0.0024∼0.0159	0.0118∼0.0808	0.0231∼0.1650
100 km	0.0017∼0.0111	0.0082∼0.0566	0.0162∼0.1154

It can be obtained from Table 3 that as the altitude of the aircraft increases, the change in the elevation angle error caused by temperature decreases. As the apparent direction of the aircraft and the deflection angle relative to the vertical line of the earth increase, the change in the elevation angle error caused by temperature increases. For every 1 K change in temperature, the elevation Aagle error changes approximately by the order of 10^−3^–10^−2^ arcseconds. For every 10 K change in temperature, the elevation angle error can vary by the order of 10^−1^ arcsecond. Through calculation, it is found that even if the temperature variation is 10 K, the influence on the accuracy of the elevation angle error is still less than 5%.

### The influence of latitude on elevation angle error

Select one representative area from each of the low-latitude, mid-latitude and high-latitude land areas in the Northern Hemisphere of the Earth to calculate the elevation angle error. The specific information is as follows: The low-latitude region is selected as East Kalaman Province of Indonesia ($1^{\circ }$N, 1$15^{\circ }$E), the mid-latitude region is selected as Beijing (40°N, 116°E), and the high-latitude region is selected as Yakute Region of Russia (70°N, 116°E). Under the conditions of calculating the elevation angle of 5–30° respectively, The elevation angle errors of the aircraft at flight altitudes of 40 km, 70 km, and 100 km are shown in Table 4. In addition to the geographical longitude and latitude, other environmental parameters are as follows: time 12:00:00 on September 1, 2022 (UTC), solar activity index F107 is 60 sfu, geomagnetic activity index Ap is 10 nT, and wavelength 0.58 *μ*m. It can be obtained from Table 2.17 that it is found that the elevation angle error decreases with the increase of latitude. This is because in regions with high latitudes, the air pressure is high, the temperature is low, and the air is thin. Compared with the middle and low latitudes, the refraction effect is not particularly significant.

**Table 4:** Comparison of elevation angle errors in low, medium and high latitude regions. (Unit: arcseconds).

Altitude	Elevation angle	East Kalaman	Beijing	Yakute
40 km	$5^{\circ }$	1.00938	1.00538	1.00329
	$10^{\circ }$	2.03433	2.02627	2.02205
	$15^{\circ }$	3.09138	3.07912	3.07272
	$20^{\circ }$	4.19915	4.1825	4.17381
	$25^{\circ }$	5.37975	5.35843	5.34728
	$30^{\circ }$	6.66072	6.63432	6.62051
70 km	$5^{\circ }$	0.58615	0.58488	0.58315
	$10^{\circ }$	1.18134	1.17878	1.17528
	$15^{\circ }$	1.79518	1.79129	1.78596
	$20^{\circ }$	2.43846	2.43318	2.42594
	$25^{\circ }$	3.12404	3.11726	3.10799
	$30^{\circ }$	3.86789	3.8595	3.84802
100 km	$5^{\circ }$	0.40986	0.40897	0.40773
	$10^{\circ }$	0.82603	0.82425	0.82175
	$15^{\circ }$	1.25524	1.25254	1.24874
	$20^{\circ }$	1.70504	1.70137	1.69621
	$25^{\circ }$	2.18441	2.17971	2.1731
	$30^{\circ }$	2.70453	2.69871	2.69052

### The influence of seasons on elevation angle error

In order to analyze the influence of seasons on elevation angle errors, the elevation angle errors in the Beijing area on April 15, 2021 (spring), July 15, 2021 (summer), October 15, 2021 (autumn), and January 15, 2022 (winter) were discussed. It is found that the elevation angle error is the largest in winter, the smallest in summer, and moderate in spring and autumn. However, as the altitude increased, the elevation angle error gradually increased in summer and gradually decreased in winter, as shown in Table 5. This is because the temperature is low in winter and the atmosphere contracts. At lower altitudes, the atmospheric density in winter is greater than that in summer, and the refraction effect is more obvious. However, as the altitude increases, the atmospheric density in winter decreases rapidly compared to summer, resulting in a gradual decrease in the altitude error in winter compared to summer.

**Table 5:** Seasonal variation of elevation angle error. (Unit: arcseconds).

Altitude	Elevation angle	Spring	Summer	Autumn	Winter
40 km	$5^{\circ }$	1.00706	1.00554	1.00668	1.00718
	$10^{\circ }$	2.02965	2.02659	2.02888	2.02988
	$15^{\circ }$	3.08426	3.07961	3.0831	3.08462
	$20^{\circ }$	4.18948	4.18317	4.18791	4.18997
	$25^{\circ }$	5.36736	5.35928	5.36534	5.36798
	$30^{\circ }$	6.64537	6.63537	6.64288	6.64613
70 km	$5^{\circ }$	0.58475	0.58486	0.58484	0.5847
	$10^{\circ }$	1.17851	1.17873	1.17869	1.17841
	$15^{\circ }$	1.79087	1.7912	1.79115	1.79072
	$20^{\circ }$	2.43261	2.43306	2.43299	2.43241
	$25^{\circ }$	3.11654	3.11712	3.11702	3.11628
	$30^{\circ }$	3.8586	3.85932	3.8592	3.85828
100 km	$5^{\circ }$	0.40883	0.40898	0.4089	0.40874
	$10^{\circ }$	0.82397	0.82427	0.8241	0.82378
	$15^{\circ }$	1.2521	1.25256	1.2523	1.25181
	$20^{\circ }$	1.70078	1.7014	1.70105	1.70038
	$25^{\circ }$	2.17896	2.17975	2.1793	2.17844
	$30^{\circ }$	2.69777	2.69875	2.6982	2.69714

### Calculation result of elevation angle error

**Fig 2:**
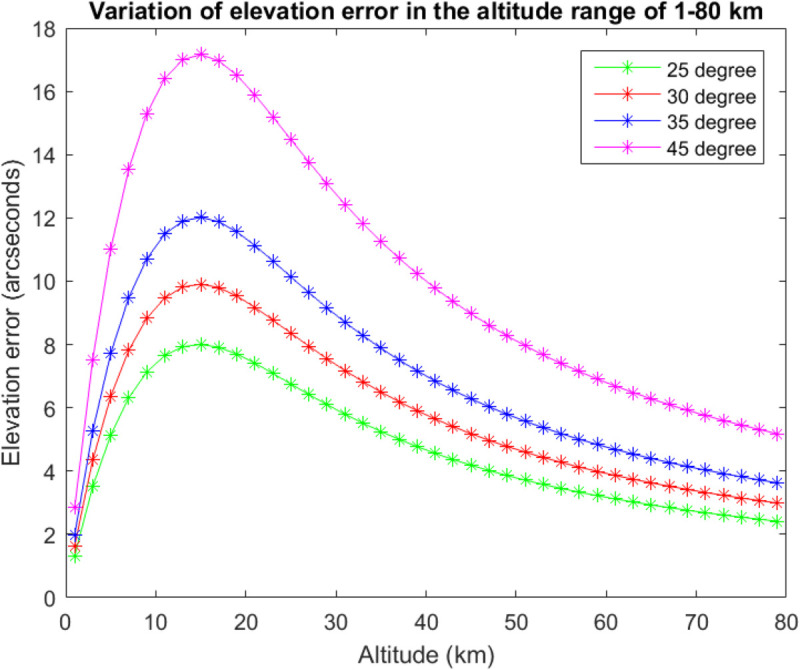
The variation law of elevation angle error with height.

By calculating the variation law of the elevation angle error with height within the altitude range of 1–80 km under the conditions of 25°, 30°, 35° and 45° respectively at the apparent zenith distance, it is found that the elevation angle error first increases and then decreases with height, as shown in Fig 2. The elevation angle error is the largest at an altitude of approximately 15 km.

## 4 Discussion, limitations, and future applications

The developed hybrid model offers a reliable and computationally practical solution for compensating atmospheric refraction errors, effectively improving geo-location accuracy in airborne and satellite remote sensing under typical atmospheric conditions. A principal strength of this approach is its combination of the Hopfield and e-index models, which strikes an effective balance between physical fidelity and operational ease, all without necessitating dedicated hardware adjustments. Nevertheless, the model’s effectiveness is bounded by several constraints. Its reliability depends heavily on the accuracy of input atmospheric profiles, such as those derived from models like NRLMSIS 2.0. Performance may degrade in situations involving highly irregular or rapidly changing conditions—including extreme weather phenomena, pronounced atmospheric ducting, or over complex terrain where the assumption of spherical stratification and horizontal homogeneity breaks down. In these demanding scenarios, more sophisticated methods incorporating data-assimilative numerical weather prediction with comprehensive three-dimensional ray-tracing would be better suited, though requiring substantially greater computational resources. Overcoming these constraints through the incorporation of real-time meteorological inputs and refined stratification methodologies constitutes an important objective for subsequent research.

Our model is designed for integration into both real-time and post-processing systems. For live ground object detection, the compensation algorithm can be embedded directly into the onboard processing units of platforms like Synthetic Aperture Radar (SAR) satellites or high-altitude reconnaissance drones, where it would utilize real-time telemetry (altitude, look-angle) and pre-loaded or forecast atmospheric profiles to apply instantaneous corrections to the geolocation data. Alternatively, for applications where the highest possible accuracy is paramount, it functions effectively as an offline correction system in ground stations, processing raw data with precisely measured atmospheric conditions. This flexibility allows for its assimilation into a wide range of Earth observation systems, including environmental monitoring satellites, disaster early warning networks, and precision mapping aircraft, significantly enhancing their target positioning accuracy without requiring hardware modifications.

## 5 Conclusion

We propose an atmospheric refraction error compensation model for ground object detection. By stratifying the atmosphere at intervals of 100 meters and combining the Hopfield refractive index model and the e-index model, the atmospheric refractive index profile is accurately simulated. Based on Snell’s law and the observation parameters of the aircraft, the error model of the true elevation angle and the apparent elevation angle is derived. The research finds that the influence of vapor pressure on the elevation angle error can be ignored (<0.005%). Every 10 K change in temperature will lead to a maximum error of 0.28 arcseconds, and the error decreases with the increase of latitude. The elevation angle error reaches its peak at an altitude of 15 kilometers, showing a vertical distribution pattern that first increases and then decreases. This model provides an error correction scheme without mechanical structural adjustment for synthetic aperture radar and remote sensing detection systems, significantly improving the positioning accuracy of surface targets, and is applicable to fields such as environmental monitoring and disaster early warning.
